# Microstructure Characterization and Interfacial Reactions between Au-Sn Solder and Different Back Metallization Systems of GaAs MMICs

**DOI:** 10.3390/ma13061266

**Published:** 2020-03-11

**Authors:** Na Wu, Yongfang Hu, Shufeng Sun

**Affiliations:** 1 Key Lab of Industrial Fluid Energy Conservation and Pollution Control, Qingdao University of Technology, Qingdao 266520, China; 2 School of Mechanical and Automotive Engineering, Qingdao University of Technology, Qingdao 266520, China; 3 14th Institute of China Electronics Technology Group Corporation, Nanjing 210000, China

**Keywords:** Au-Sn, die bonding, metallization system, microstructure, interfacial reaction

## Abstract

GaAs monolithic microwave integrated circuits (MMICs) with different back metallization systems (TiW/Au and Au/Ti/Au) exhibit different problems in the automatic Au-Sn eutectic bonding process, such as edge breakage or excessive voids. In this study, the formation mechanism of the edge breakage and excessive voids were investigated to prevent the damage of the MMICs in mass production scenarios. The microstructure and elemental distribution were studied using a scanning electron microscope and energy-dispersive spectroscopy. The void contents of the brazed region were measured with three-dimensional computed tomography. The top Au layer of the TiW/Au metallization partially dissolved in the melting An-Sn solder. Consequently, liquidus temperature of the solder increased, leading to isothermal solidification with the formation of ζ-Au_5_Sn in the scrubbing process, which was the reason for the edge breakage. The terminal Au film of the Au/Ti/Au metallization completely dissolved in the melting An-Sn solder. The metallurgical combination was achieved by the formation of the TiAu_4_ intermetallic compound between the Au-Sn solder and the Ti layer. The wettability of Au-Sn solder on Ti layer should be improved to prevent the formation of the excessive voids.

## 1. Introduction

In recent decades, monolithic microwave integrated circuit (MMIC) amplifiers based on GaAs pseudomorphic high electron mobility transistor (PHEMT) technology have been playing an essential role in satellite telecommunications and radar applications [1]. Due to the continuous downscaling of PHEMTs and the low thermal conductivity of GaAs, thermal issues in MMIC amplifiers have become increasingly significant [2]. The primary solution to relieving the high heat flux issue is to distribute the thermal energy onto a larger area, which requires die bonding and a submount with high thermal conductivity [3,4].

Au80-Sn20 solder exhibits excellent electrical and thermal conductivity, good high-temperature performance, outstanding fatigue and creep resistance, and superior corrosion resistance [5,6,7,8,9]. Moreover, Au-Sn solder provides the additional benefit of not requiring flux during reflow, significantly reducing the potential for contamination and pad corrosion [10]. Consequently, it is increasingly utilized in environments requiring high-frequency, highly reliable, and high-temperature applications.

Furthermore, metallization is critical to advanced packaging technologies, since it enhances the wetting and spreading of difficult-to-solder base materials [11]. The back metallization systems of GaAs MMICs used in this research were TiW/Au and Au/Ti/Au. In the automatic die-bonding production, edge breakage would occur in the MMIC with TiW/Au system, invalidating the MMICs. Excessive voids would appear in the brazed region of the Au/Ti/Au system, preventing the heat flux transfer. Thus, the production throughput was at a low level. So, it is important to find the formation mechanism of the edge breakage and excessive voids to prevent damage occurring to the MMICs. 

However, the present study is mainly focused on the interfacial reactions between Au-Sn solder and metallizations, such as Ni [12,13,14,15,16], Cu [16,17,18,19], Ag [19], Pt [16], Ti/Ni/Au, Cr/Ni/Au [20], and Ti/Pt/Au [21]. The interfacial products mainly included Ni_3_Sn, Ni_3_Sn_2_, (Au, Ni)Sn, (Au, Ni)_3_Sn_2_, (Au, Ni)_5_Sn, AuCu_3_, (Au, Cu)_5_Sn, and Ag_3_Sn. There are a few reports on the interfacial reactions between TiW/Au, Au/Ti/Au, and Au-Sn solder. In this study, GaAs MMICs with different back metallization systems were brazed to Mo/Cu submount using Au80-Sn20 eutectic solder. The interfacial reactions between the Au-Sn solder and the back metallization systems, as well as the bonding behavior, were studied to ascertain the formation mechanism of the edge breakage and excessive voids to improve the Au-Sn eutectic bonding process.

## 2. Materials and Methods

Two types of GaAs MMICs (CETC Co., Beijing, China) with TiW/Au and Au/Ti/Au back multilayer metallization systems were used, both of which were terminated with an Au layer with thicknesses of 6 and 0.2 µm, respectively. The 6 µm thick Au layer was electrodeposited, and the 0.2 µm thick Au layer was deposited in an industrial-scale sputtering chamber.

The highly thermally-conductive submount used in this study was Mo/Cu alloy with Ni/Au (5 µm/3 µm) metallization. The Mo/Cu alloy was produced by powder sintering, and the composition was Mo70-Cu30 in weight percent. Mo and Cu powder were mixed according to their weight percent ratio (7:3), and compressed into a green compact with a thickness of 3.0 mm. The green compact was sintered in a reducing atmosphere at 1350 °C for 4 h. The workpiece was preheated to 100 °C and rolled into the desirable thickness using a roller at 50 °C. A Ni layer was electrodeposited from a sulfamate plating solution (nickel aminosulfonate 420 mL/L, NiCl_2_·6H_2_O 10 g/L, and H_3_BO_3_ 37.5 g/L) at 55 °C and a current density of 0.8 A/dm^2^. A Au layer was electrodeposited from a cyanide plating solution (KAu(CN)_2_ 15 g/L, K_2_HPO_4_ 40 g/L, and KH_2_PO_4_ 10 g/L) at 60 °C with current density of 0.4 A/dm^2^. Nickel aminosulfonate was supplied by Atotech Co. (Berlin, Germany). The other chemicals used were analytically pure and commercially available.

Au80-Sn20 (wt.%) eutectic solder with a melting point of 280 °C was used to bonding the GaAs MMIC to the Mo/Cu submount. The initial Au-Sn foil with a thickness of 25 µm was supplied by Source Co. (GuangDong, China). and offered in the form of preform. The physical characteristics of materials used in this study are shown in Table 1.

The surfaces of Mo/Cu submount and Au-Sn preform were cleaned by Ar plasma before soldering. Since the MMICs were vacuum sealed in Gel-Pak boxes in the purification factory, they were used in the as-supplied state.

**Figure 1:**
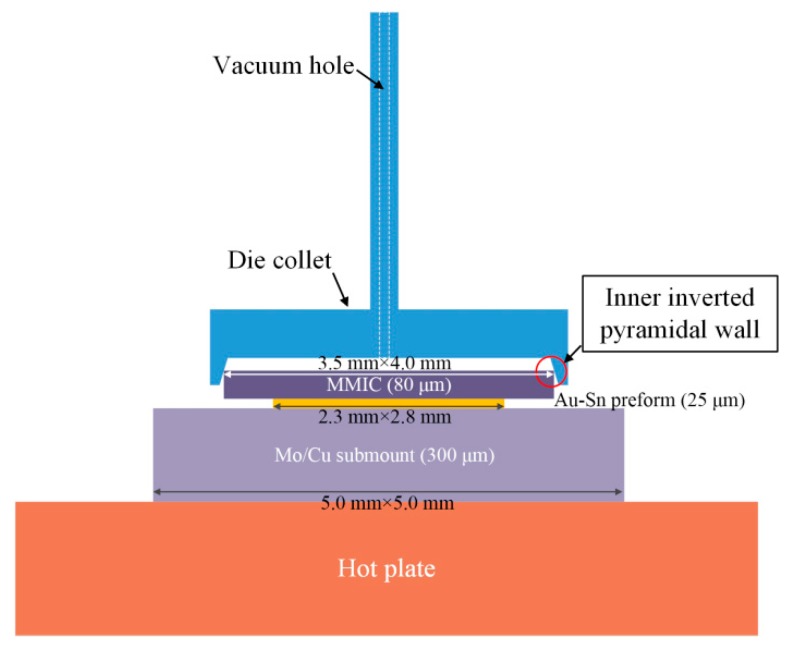
Schematic diagram of Au-Sn eutectic bonding.

The Au-Sn eutectic soldering process was automatically carried out using a die bonder machine (Figure 1). The geometrical parameters of the Mo/Cu submount, Au-Sn preform, and the MMIC were 5.0 mm × 5.0 mm × 300 µm, 2.8 mm × 2.3 mm × 25 µm, and 3.5 mm × 4.0 mm × 80 µm, respectively. First, the Mo/Cu submount was picked up and placed on the hot plate using a vacuum nozzle by contacting its surface. The magnitude of the vacuum was 500 mm-Hg. Second, the Au-Sn preform was picked up and placed on the submount in the same way. Lastly, the pickup and placement of the MMIC was achieved via a vacuum die collet. The die collet was customized from the professional pick tools company according to the MMIC size. There were inner inverted pyramidal walls in the die collet. The inner inverted pyramidal walls were used to hold the MMIC by contacting the edges rather than directly contacting the surface, which avoided damaging the sensitive surface of the MMIC.

**Figure 2:**
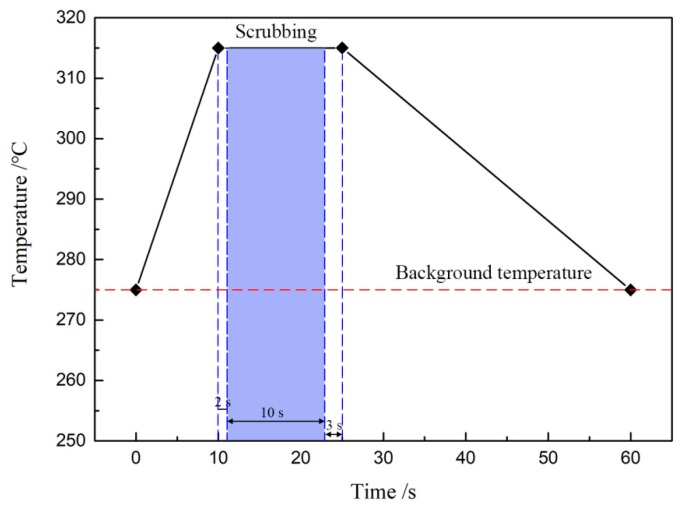
Technological process of Au-Sn eutectic bonding.

Figure 2 shows the technological process of Au-Sn eutectic bonding. As the temperature increased to 315 °C, the die collet was still holding the MMIC. Since the die collet was manufactured with tungsten carbide, it could stand the high soldering temperature. The bond head of the die bonder drove the die collet to move the MMIC according to the preset contact force and scrubbing pattern. This was called the “scrubbing” process, promoting the melting Au-Sn solder to spread, as the filled column in Figure 2 illustrated. The scrubbing process was started several seconds after the temperature reached 315 °C. It was for this reason that the Au-Sn solder could melt sufficiently and exhibited better wettability, which led the solder to spread and wet the base materials in the scrubbing process. There were also several seconds between the finish of scrubbing and start of the temperature decrease. If the scrubbing process continued to the end of the holding stage, an excess amount of Au-Sn solder would spread to the area around the MMIC and the area under the MMIC would lack Au-Sn solder. The time was extended to promote elemental diffusion and reactions between Au-Sn solder and base materials.

The void contents in the Au-Sn brazed region were measured by three-dimensional computed tomography (3D CT) on Nikon inspection system (Tokyo, Japan). Specimens for metallographic examinations were sectioned from the solder joints. The cross sections were ground, polished, and etched with a solution of 1.25 g I_2_ + 1.25 g KI dissolved in 1.25 mL H_2_O and 85 mL C_2_H_5_OH at room temperature for 30–60 s. All the chemicals were of commercial grade and analytically pure. The microstructure and elemental distribution were studied using an scanning electron microscope (SEM, FEI, Oregon, USA) and energy-dispersive spectroscopy (EDS, OXFORD, Oxford, UK).

## 3. Results and Discussion

### 3.1. TiW/Au Metallization

**Figure 3:**
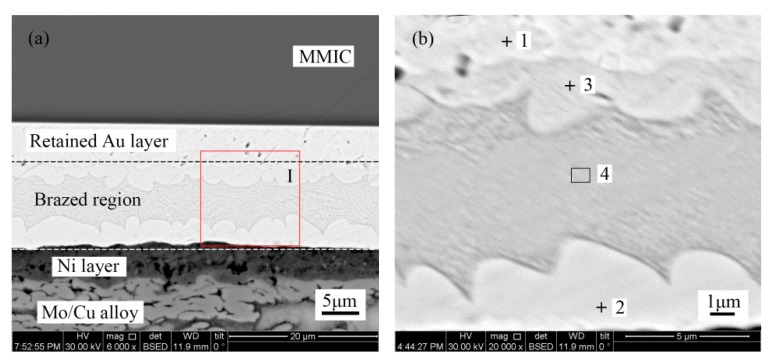
(**a**) Microstructure of the TiW/Au|Au-Sn eutectic joint and (**b**) region I in higher magnification.

Figure 3 shows the microstructure of the Au-Sn solder joint of the TiW/Au metallization system. The brazed region was divided into four distinct regions, as denoted in Figure 3b, while Table 2 shows the chemical composition of each region measured with EDS.

Point 1 in the region near the MMIC mainly consisted of Au with a small amount of Ga and As. Therefore, the region was considered to be the retained Au of the terminal metalized Au layer. The initial thickness was about 6 µm, while the retained thickness was about 4–5 µm. It was suggested that a 1–2 µm Au layer dissolved into the Au-Sn solder during the bonding process.

**Figure 4:**
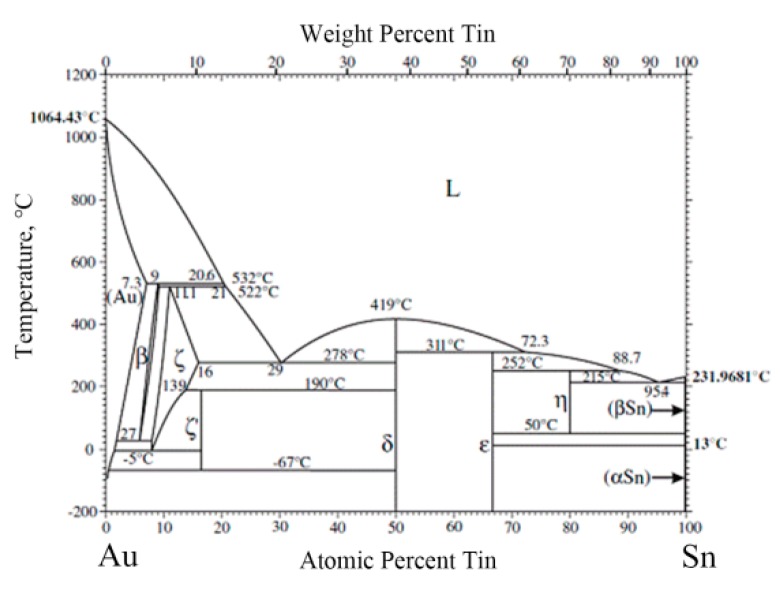
Au-Sn phase diagram [22].

The Au/Sn ratio of point 2 close to Mo/Cu alloy was nearly 5, with some Ni. Therefore, it was determined to be ζ-(Au,Ni)_5_Sn, according to the Au-Sn phase diagram (Figure 4) [22]. Compared with the initial coating of Mo/Cu alloy, the Au layer completely dissolved into the melting Au-Sn solder. A metallurgical combination occurred between the Au-Sn solder and the Ni metallization layer. The same principle was applied to point 3 near the retained Au layer, which was identified as ζ-Au_5_Sn.

As the Au-enriched Au_5_Sn intermetallic compound (IMC) solidified, the Au content declined and the Sn content increased in the remaining melt. When the temperature dropped to 280 °C, the eutectic reaction L→δ-Au-Sn + ζ-Au_5_Sn occurred with the formation of Au-Sn eutectic. Furthermore, considering the morphology and composition of region 4, it was determined to be an Au-Sn eutectic. Therefore, the region between the MMIC and the Mo/Cu alloy was divided into the retained Au layer region and the brazed region (Figure 3a).

**Figure 5:**
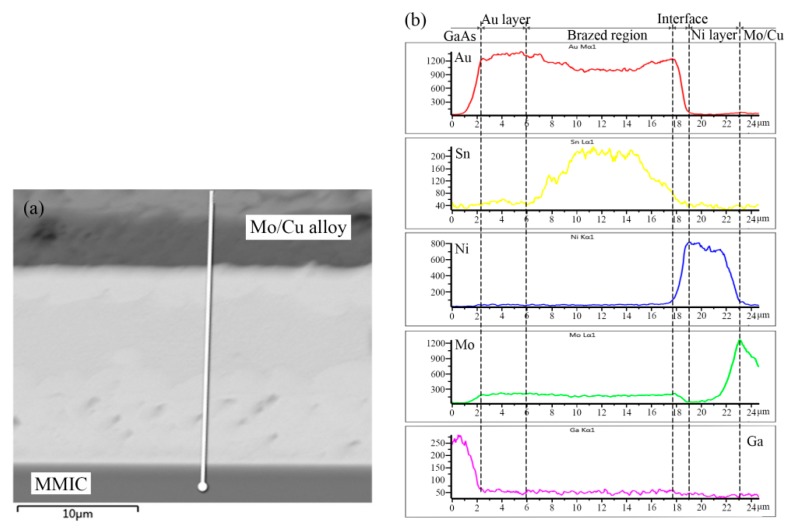
EDS analysis of TiW/Au|Au-Sn eutectic joint: (**a**) measured position and (**b**) elemental distribution.

The elemental distribution across the TiW/Au|Au-Sn eutectic joint was analyzed using EDS ( OXFORD, Oxford, UK). The measured position and results are shown in Figure 5.

The distribution of Au from the brazed region to the back Au layer of MMIC increased gradually, while the distribution of Sn presented a steep decrease. A metallurgical combination of the brazed region to MMIC was mainly achieved through the partial dissolution of the back Au layer in the melting Au-Sn solder.

A transition zone from the brazed region to the Ni metallization layer of the Mo/Cu alloy was evident, displaying a noticeable increase in Ni and a decrease in Au. According to the microstructure, the transition zone consisted of ζ-(Au,Ni)_5_Sn IMC. Since the peak heating temperature was maintained for less than 30 s, the ζ-(Au,Ni)_5_Sn interface was only 1.5 µm thick. Joining of the brazed region to Mo/Cu alloy was realized by the diffusion between the Ni metallization layer and the Au-Sn solder. The Au metallization layer prevented the oxidation of the Ni layer before soldering.

A noticeable peak of Sn appeared in the center of the brazed region, while a valley was evident at each side of the brazed region. Therefore, Au-enriched ζ-Au_5_Sn was initially formed during the solidification process, whereas Sn was segregated in the residual liquid.

### 3.2. Au/Ti/Au Metallization

**Figure 6:**
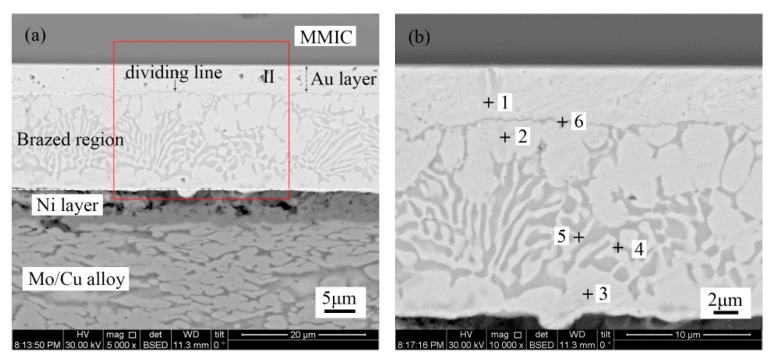
(**a**) Microstructure of the Au/Ti/Au|Au-Sn eutectic joint and (**b**) region II in higher magnification.

Figure 6 shows the microstructure of the Au-Sn solder joint of the Au/Ti/0.2 µm Au metallization system. It is evident that the TiW/Au|Au-Sn solder joint displayed a distinct dividing line in the brazed region. Table 3 shows the chemical composition of each area measured with EDS.

The concentration of Au was as high as 96.2% at point 1 close to MMIC. Therefore, this region was the bottom Au layer of the Au/Ti/0.2 µm Au metallization.

The region (point 2) next to the bottom Au layer consisted of Au (75.5%) and Sn (14.7%). According to the Au-Sn phase diagram, it was determined to be ζ-Au_5_Sn. Similar to the TiW/Au|Au-Sn solder process, the region near to Mo/Cu alloy was ζ-(Au,Ni)_5_Sn. The top Au layer completely dissolved into Au-Sn solder and the brazed region metallurgically combined with the Ni layer.

The center of the brazed region displayed a chrysanthemum-like structure, consisting of two phases (point 4 and point 5). According to the chemical composition and Au-Sn diagram, the two phases were ζ-Au_5_Sn and δ-Au-Sn. The brazed region was composed of ζ-Au_5_Sn at both sides and an Au-Sn eutectic in the center.

**Figure 7:**
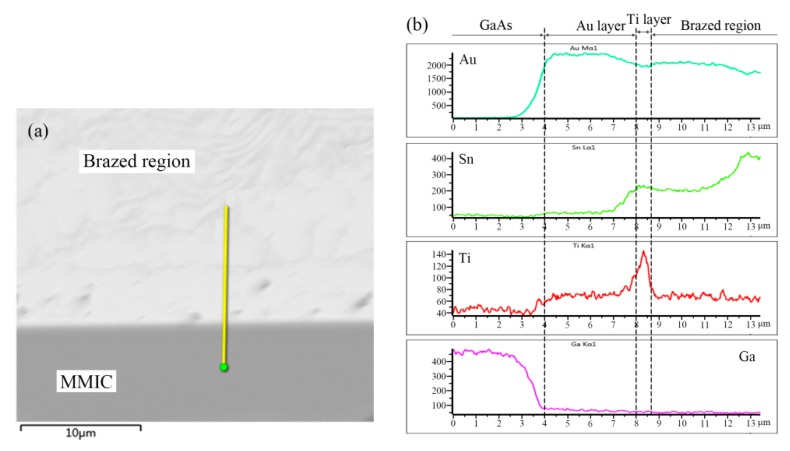
EDS analysis of the Au/Ti/Au|Au-Sn eutectic joint: (**a**) measured position and (**b**) elemental distribution.

EDS analysis indicated the presence of element Ti in the dividing line. Furthermore, elemental distribution across the Au/Ti/Au|Au-Sn eutectic joint was measured; the results are shown in Figure 7.

A distinct peak appeared in the elemental distribution of Ti. Therefore, the dividing line was identified as the Ti layer of the Au/Ti/Au metallization system. The top 0.2 µm Au layer completely dissolved in the Au-Sn solder, whereas the Ti acted as a diffusion barrier layer preventing dissolution of the bottom Au layer. The combination of the Au/Ti/0.2 µmAu metallization system and the Au-Sn solder occurred through dissolution of the top Au layer into the Au-Sn, and directly exposed the Ti layer to the Au-Sn solder. The melting Au-Sn solder wetted the Ti layer and the interconnection was established by the formation of TiAu_4_ IMC interface [23].

### 3.3. Interfacial Reactions

Schematic diagrams of the microstructure evolution in the soldering processes were drawn according to the microstructure and interfacial reactions of the solder joints.

**Figure 8:**
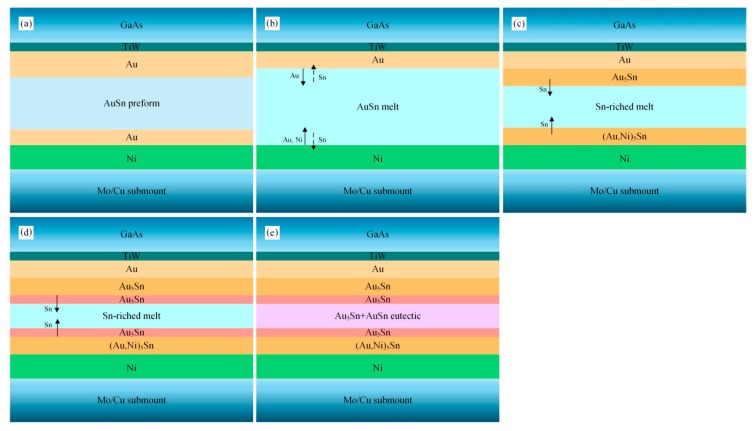
Schematic diagram of the microstructure evolution process of the TiW/Au|Au-Sn joint: (**a**) initial state, (**b**) dissolution and diffusion between the metallization system and the Au-Sn solder, (**c**) isothermal solidification with the formation of Au_5_Sn, (**d**) nonisothermal solidification with the formation of Au_5_Sn, and (**e**) nonisothermal solidification with the formation of Au-Sn eutectic.

Figure 8 shows the schematic diagram of the microstructure evolution of the TiW/Au|Au-Sn joint. The Au-Sn solder started to melt when the reflow temperature reached 280 °C. The terminal metalized 6 µm Au layer on the back of the MMIC partially dissolved into the melting Au-Sn solder, whereas the 3 µm Au layer of the Ni/Au metallization on the Mo/Cu submount completely dissolved into the melting Au-Sn solder. Therefore, the Ni layer was directly exposed to the melting Au-Sn solder (Figure 8b).

Consequently, the Au content in the melting solder increased and deviated from the eutectic point, resulting in increase of the liquidus temperature. When the liquidus temperature increased to the holding temperature, isothermal solidification occurred. The melting solder (*L*) transformed into solid–liquid phase mixtures: *L*→ζ + *L’* (Sn-enriched melt). The Au-enriched ζ-Au_5_Sn and ζ-(Au, Ni)_5_Sn were formed by attaching to the retained Au layer of MMIC and the Ni layer of the Mo/Cu submount, respectively (Figure 8c).

Sn was progressively enriched in the residual liquid as the isothermal solidification proceeded. When the holding stage ended, the brazing temperature started to decrease inducing nonisothermal solidification. The ζ-Au_5_Sn precipitated in the residual liquid (Figure 8d), and the Sn content further increased. When the composition of the residual liquid reached the eutectic point, the eutectic reaction *L’*→δ-Au-Sn + ζ-Au_5_Sn occurred with the formation of the Au-Sn eutectic (Figure 8e).

**Figure 9:**
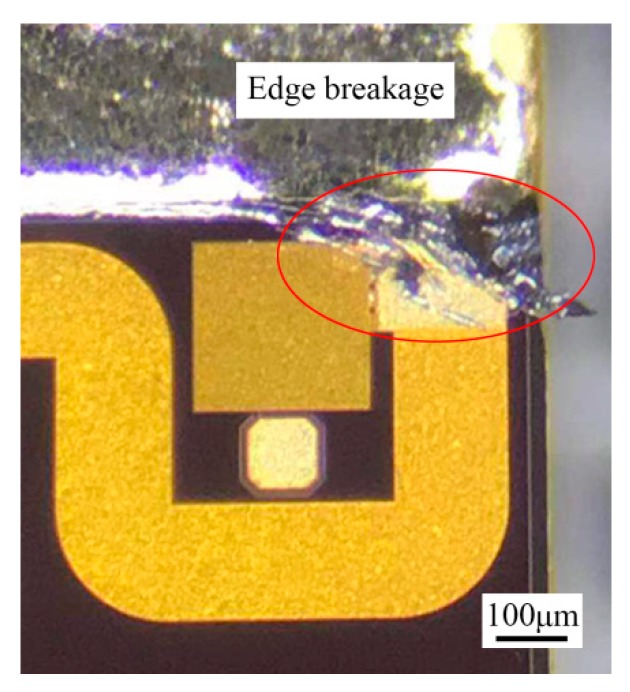
Edge breakage of GaAs MMIC in the TiW/Au|Au-Sn eutectic joint.

A deviation of 1 wt.% Au from the eutectic composition towards the Au-rich side results in an approximately 30 °C increase in the liquidus temperature [21]. Due to the uneven spreading of the filler metal, some area of the brazed region locally lacked melting Au-Sn solder. The Au layer dissolved into the melting solder, resulting in an obvious local increase of Au. Therefore, isothermal solidification occurred through the local brazed region. The solder seemed to be “frozen” during the scrubbing process, and edge breakage of the MMIC was caused by the still-moving die collet (Figure 9). Therefore, a larger and thinner preform was preferred to avoid the local lack of Au-Sn solder.

**Figure 10:**
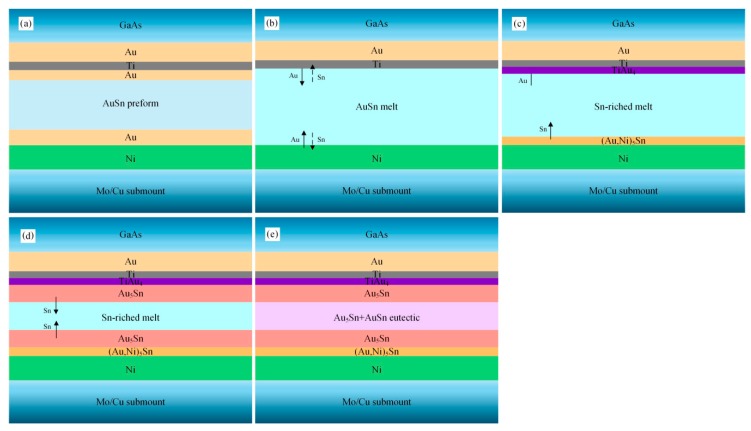
Schematic diagram of the microstructure evolution process of the Au/Ti/Au|Au-Sn joint: (**a**) initial state, (**b**) dissolution and diffusion between the metallization system and the Au-Sn solder, (**c**) formation of TiAu_4_, (**d**) nonisothermal solidification with the formation of Au_5_Sn, and (**e**) nonisothermal solidification with the formation of Au-Sn eutectic.

Figure 10 shows a schematic diagram of the microstructure evolution process of the Au/Ti/Au|Au-Sn joint. The terminal Au layer (0.2 µm) of the Au/Ti/Au metallization system dissolved into the melting Au-Sn solder (Figure 10b). The Ti layer acted as a diffusion barrier layer, preventing the bottom Au layer from dissolving. The melting solder wetted and reacted with the Ti layer with the formation of TiAu_4_ IMC (Figure 10c). The thin, continuous IMC layer was essential for effective bonding.

Compared with the TiW/Au metallization system, significantly less Au dissolved in the Au-Sn solder. Therefore, a short, or even no, isothermal solidification process was induced. The nonisothermal solidification process was similar to that of the TiW/Au|Au-Sn eutectic joint (Figure 10d,e).

**Figure 11:**
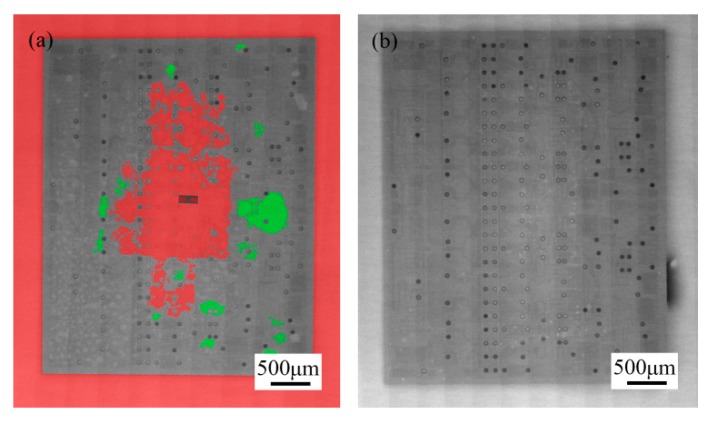
Void content of the Au/Ti/Au|Au-Sn eutectic joint: (**a**) 18.54% and (**b**) 2.36%.

**Figure 12:**
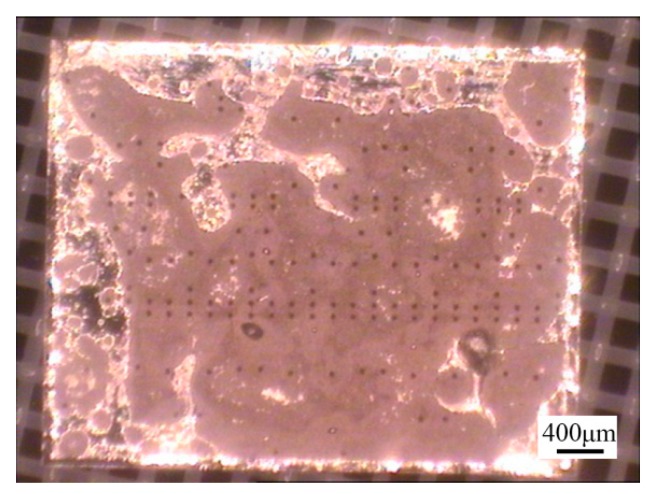
The MMIC sample in Figure 11a separated by “hot-lifting”.

**Figure 13:**
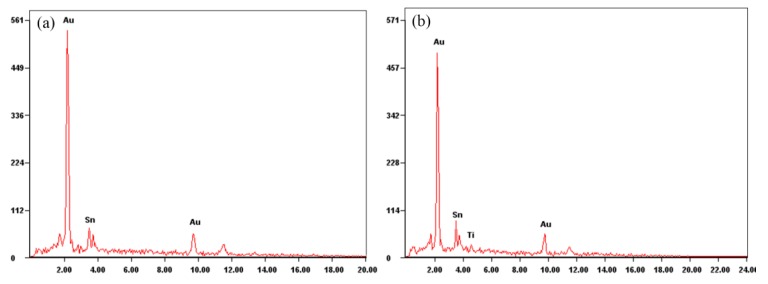
EDS analysis of the soldered MMIC after separation: (**a**) wetted area and (**b**) unwetted area.

However, 3D CT revealed the presence of a large number of voids in the brazed region (Figure 11). The samples with a high void content were separated by “hot-lifting” on a hot plate. A broad gray area of the MMIC was not wetted by the Au-Sn solder (Figure 12). The chemical compositions of the wetted and unwetted areas were detected by EDS (Figure 13). Compared with the wetted area, the unwetted area contained Ti besides the Au and Sn. Thus, the excessive voids were the reason that the Ti layer was not wetted by the Au-Sn solder. The oxidation film prevented the melting of Au-Sn solder wetting Ti layer [24]. Therefore, a small specific surface area of the preform was preferred to improve the wettability. The void content can be decreased to 2.36% (Figure 11b).

## 4. Conclusions

GaAs MMICs with different back metallization systems (TiW/Au and Au/Ti/Au) were Au-Sn eutectic bonded in the mass production. The microstructure characterization and interfacial reactions during the bonding process were studied to ascertain the formation mechanism of the edge breakage and excessive voids.

1. The terminal metalized Au layer of the TiW/Au metallization partially dissolved in the melting An-Sn solder. Isothermal solidification locally occurred with the formation of ζ-Au_5_Sn in the scrubbing process, inducing the edge breakage of the MMIC. A larger and thinner preform was preferred to avoid the local lack of Au-Sn solder.
2. The top Au film of the Au/Ti/Au metallization completely dissolved in the melting An-Sn solder. The combination was achieved by the wetting and reaction of Au-Sn solder to the Ti layer with the formation of TiAu_4_ IMC. The oxidation film of Au-Sn preform prevented the melting solder wetting Ti layer. A small specific surface area of the preform was favored to improve the wettability.

## Figures and Tables

**Table 1:** Physical characteristics of materials used in this study.

Material	Density (g·cm^−3^)	Melting Point (°C)	Thermal Conductivity (W·m^−1^·K^−1^)	Coefficient of Thermal Expansion (10^−6^ K^−1^)
GaAs	5.37	1238	39	5.8
Au80-Sn20	14.5	280	57	16
Mo70-Cu30	9.8	—	180–200	9.1

**Table 2:** Chemical composition of each region in the TiW/Au|Au-Sn eutectic joint.

Position	Element Composition (at.%)						Phase
	Au	Sn	Ni	Cu	Ga	As	
Point 1	92.6	—	—	—	4.8	2.6	Retained Au
Point 2	79.7	13.3	5.5	1.5	—	—	ζ-(Au,Ni)_5_Sn
Point 3	86.1	13.9	—	—	—	—	ζ-Au_5_Sn
Region 4	70.8	29.2	—	—	—	—	Au-Sn eutectic

**Table 3:** Chemical composition of each region in the Au/Ti/Au|Au-Sn eutectic joint.

Position	Element Composition (at.%)							Phase	
	Au	Sn	Ni	Mo	Cu	Ga	Ti		
Point 1	96.2	—	—	—	—	3.8	—	Bottom Au layer	
Point 2	83.7	16.3	—	—	—	—	—	ζ-Au_5_Sn	
Point 3	75.5	14.7	4.7	3.7	1.5	—	—	ζ-(Au,Ni)_5_Sn	
Point 4	71.3	23.0	1.2	3.2	0.9	0.5	—	Au_5_Sn	Au-Sn eutectic
Point 5	58.4	39.4	—	2.2	—	—	—	Au-Sn	
Point 6	77.0	14.4	—	—	—	—	8.6	TiAu_4_	
